# Factors Associated with Dental Service Use Based on the Andersen Model: A Systematic Review

**DOI:** 10.3390/ijerph18052491

**Published:** 2021-03-03

**Authors:** André Hajek, Benedikt Kretzler, Hans-Helmut König

**Affiliations:** Department of Health Economics and Health Services Research, University Medical Center Hamburg-Eppendorf, 20251 Hamburg, Germany; b.kretzler.ext@uke.de (B.K.); h.koenig@uke.de (H.-H.K.)

**Keywords:** dental service, dental visits, dental care use, dental care utilization, oral health services, Andersen model, dental service use, systematic review, inequality, dental services research, health services research, health care use, health care utilization, access to dental services

## Abstract

Background: A systematic review synthesizing studies examining the determinants of dental service use drawing on the (extended) Andersen model is lacking. Hence, our purpose was to fill this knowledge gap; Methods: Three established electronic databases (PubMed, PsycInfo, as well as CINAHL) were searched. Observational studies focusing on the determinants of dental service use drawing on the Andersen model were included; Results: In sum, 41 studies have been included (ten studies investigating children/adolescents and 31 studies investigating adults). Among children, particularly higher age (predisposing characteristic), higher income (enabling resource) and more oral health problems (need factor) were associated with increased dental service use. Among adults, findings are, in general, less consistent. However, it should be noted that one half of the studies found an association between increased education (predisposing characteristic) and increased dental service. In general, study quality was rather high. However, it should be noted that most studies did not report how they dealt with missing data; Conclusions: Our systematic review revealed that all components (i.e., predisposing characteristics, enabling resources and need factors) of the Andersen model tend to be associated with dental service use among children, whereas the findings are more mixed among adults. In conclusion, beyond need factors, dental service use also tend to be driven by other factors. This may indicate over—or, more likely—underuse of dental services and could enrich the inequality discussion in dental services research.

## 1. Introduction

Besides hospitalization and outpatient physician visits, dental visits are an important component of health care use. An increased dental service use (all types of dental services) reflects an increased economic burden. Moreover, it has been shown that frequent use of dental services is associated with negative emotions [1] and potential overtreatment [2]. However, postponing dental visits can also have deleterious oral health [3] and well-being effects [4,5]. Therefore, knowledge about the factors associated with dental service use is important. Ultimately, this knowledge may be beneficial in managing dental service use and may help to avoid under-, over- or misuse of dental services.

Drawing on the well-known Andersen model [6], various studies have examined the determinants of hospitalization or doctor visits [7,8]. It is an important “behavioral model of health service use”. Commonly, it differentiates between predisposing characteristics like sex or age, enabling resources like perceived access to health care use or disposable income, and need factors such as chronic diseases or self-rated health.

In further detail, individual predisposing characteristics cover social factors like education or social ties or “biological factors” like sex or age. Additionally, contextual predisposing factors cover, for example, cultural norms.

Enabling resources cover financial and organizational factors which could affect use of health services. Individual financing factors cover income and wealth (e.g., to pay for health services or out-of-pocket payments). Organizational factors include, among other things, waiting time for health care, transportation or travel time. Furthermore, contextual factors cover, e.g., hospital and physician density.

It can be differentiated between individual perceived need (like subjective health) and evaluated need (like illnesses diagnosed by a physician). Contextual need factors include environmental need characteristics like traffic and population health indices like indicators of disability.

Psychosocial factors like loneliness or personality-related factors are included in the extended Andersen model [8]. Various studies have shown that particularly need factors are associated with general health care use [9].

To date, several studies [10,11,12] exist analyzing the factors associated with dental service use based on the Andersen model [6] mainly showing that predisposing characteristics, enabling resources (depending on the healthcare system) and need factors (such as oral health-related quality of life [13,14]) can determine dental service use.

Since it is often the aim of health care systems to provide equitable access to dental services, decreasing the influence of predisposing and particularly enabling resources is a key objective. To date, a systematic review is lacking synthesizing the existing evidence on the determinants of dental service use drawing on the Andersen model. Therefore, the purpose of the current systematic review is to address this gap in knowledge. In sum, this knowledge may assist in managing dental service use. In turn, this knowledge may help to increase oral health related quality of life [15]. Additionally, this systematic review may identify potential gaps in knowledge and may therefore guide and inspire future research in this area.

## 2. Materials and Methods

The methods of this review are in line with the Preferred Reporting Items for Systematic Reviews and Meta-Analysis (PRISMA) guidelines [16]. It should be noted that our work was registered to the International Prospective Register of Systematic Reviews (PROSPERO, registration number: CRD42020193094). Additionally, a systematic review protocol has been published [12].

### 2.1. Search Strategy and Selection Criteria

In September 2020, a systematic literature search (PubMed, PsycINFO, CINAHL) was conducted. The search query for PubMed is described in Table 1. Two reviewers (AH and BK) independently evaluated studies for inclusion. First, a title/abstract screening was conducted and second, a full-text screening was performed. Additionally, a manual hand search was conducted based on the references of the identified articles and also using forwards citation tracking.

If disagreements occurred, discussions were used to resolve them. This procedure was also applied if disagreements occurred in data extraction and evaluating the study quality.

For this systematic review, inclusion criteria were as follows: (1) observational studies examining the determinants of dental service use, (2) studies explicitly drawing on the Andersen model, (3) measurement of important variables with appropriate tools (e.g., using adequate tools to quantify dental service use), (4) studies in German or English language, published in a peer-reviewed journal. Studies were excluded when: (1) studies did not examine the determinants of dental service use, (2) studies did not use the Andersen model as theoretical foundation, (3) studies solely using disease-specific samples (such as individuals with cognitive disorders), (4) studies other than observational, (5) inappropriate measurement of key variables (e.g., unclear period for dental service use), (6) studies not published in English or German language or not published in a peer-reviewed scientific journal. There were no restrictions regarding location, demographic factors or time. A pretest was conducted prior to final eligibility criteria (sample of 100 titles/abstracts). However, the eligibility criteria did not change.

### 2.2. Data Extraction and Analysis

One reviewer (BK) conducted the data extraction. The data extraction was cross-checked by the second reviewer (AH). If clarification was required, the study authors were contacted. The data extraction covered study design, explanatory variables (drawing on the components of the Andersen model), assessment of dental service use, characteristics of the sample, statistical approach and main findings.

### 2.3. Quality Assessment

To date, no consensus exists on a tool to assess the quality of health care use (HCU) studies [17]. Hence, in this current work we used a HCU tool originally developed by Stuhldreher et al. [18] and refined by Hohls et al. [19]. Additional details are provided by Hajek et al. [20]. It was also used in previous research (e.g., [17,19]). The quality assessment was performed by two reviewers (AH, BK).

## 3. Results

**Figure 1:**
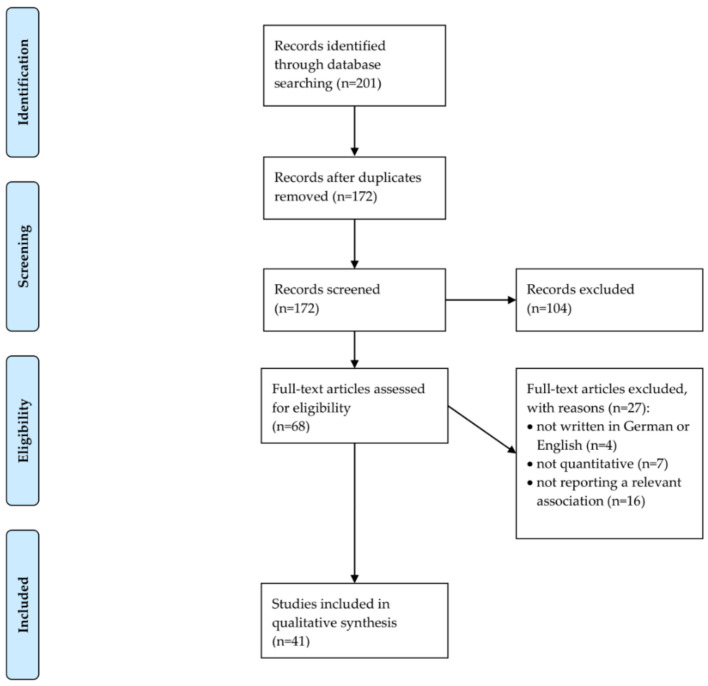
PRISMA 2009 Flow Chart.

The process of study selection is shown in Figure 1 (flow chart [21]). In sum, *n* = 41 studies were included in our final synthesis (ten studies investigating children/adolescents [22,23,24,25,26,27,28,29,30,31] and 31 studies investigating adults [32,33,34,35,36,37,38,39,40,41,42,43,44,45,46,47,48,49,50,51,52,53,54,55,56,57,58,59,60,61,62]). We will present an overview of included studies by age group (children/adolescents; adults) in the next two sections.

### 3.1. Overview of Included Studies: Children/Adolescents

An overview of the studies and key findings among children/adolescents is shown in Table 2. Results of adjusted regressions are presented in Table 2. Studies were published between the years 2007 and 2020. Data came from South America (*n* = 4 studies, with: Brazil, *n* = 3; Peru, *n* = 1), North America (*n* = 3 studies, with: United States, *n* = 2; Canada, *n* = 1), and Asia (*n* = 3, with: China, *n* = 2; Saudi Arabia, *n* = 1). Nine out of the ten studies had a cross-sectional design, and one study had a longitudinal design [30].

The period of dental service use ranged from six months [23,29] to ever visiting a dentist in one’s lifetime (in Saudi Arabia [22] and Brazil [24,25]). While all studies examined predisposing characteristics, nine out of the ten studies examined enabling resources [22,23,24,25,26,27,28,29,30], eight out of the ten studies examined need factors [22,24,25,26,27,28,30,31] and one study examined psychosocial factors [30]. The studies included covered all age groups in childhood/adolescence.

The sample size ranged from *n* = 350 individuals [24] to *n* = 71,614 individuals [23], all age groups in childhood and adolescence were covered and the proportion of female children/adolescents ranged from 41% to 58%. Most studies used data from large survey studies. More details are shown in Table 2.

### 3.2. Overview of Included Studies: Adults

An overview of the studies and key findings among adults is shown in Table 3. These studies were published between the years 1981 and 2020. Data came from Europe (*n* = 4 studies; Germany, *n* = 2; Sweden, *n* = 1; Finland, *n* = 1), South America (*n* = 6 studies, all studies were from Brazil), North America (*n* = 13 studies; United States, *n* = 12; Canada, *n* = 1), Asia (*n* = 6 studies; China, *n* = 2; South Korea, *n* = 1; Thailand, *n* = 1; Israel, *n* = 1; Sri Lanka, *n* = 1), Africa (*n* = 2 studies; Burkina Faso, *n* = 1; Sudan, *n* = 1). Three [32,39,48] out of the 31 studies had a longitudinal design, whereas the other 28 studies had a cross-sectional design [33,34,35,36,37,38,40,41,42,43,44,45,46,47,49,50,51,52,53,54,55,56,57,58,59,60,61,62].

The period of dental service use ranged from one year [33,34,35,36,37,40,41,42,44,48,50,55,57,58,59,60,61] to ever visiting a dentist in one’s lifetime (in Brazil [53]). All studies investigated predisposing characteristics, 30 out of the 31 studies examined enabling resources (except for [38]), and 29 studies examined need factors (except for [35,47]). Across the studies, the sample size ranged from 210 individuals [38] to *n* = 60,202 individuals [42], the average age ranged from 28 [54] to 78 years [59], and the proportion of women ranged from 0% [57] to 72% [45]—with most studies having a proportion of women from 40% to 70%. While several studies used data from large survey studies, a few studies used rather specific samples (e.g., employees of public sector institutions in Kandy, Sri Lanka [38] or University employees in the United States [54]). Further details are shown in Table 3.

The key findings of our review are displayed in Table 4 (children) and Table 5 (adults) (for further details, please see Tables S1 and S2). The displayed determinants stratified by the component of the Andersen model (i.e., predisposing characteristics; enabling resources; need factors; psychosocial factors) displayed in Table 4 and Table 5 were selected since they were investigated in at least half of the studies. However, it should be noted that none of the need factors met this criterion among adults (i.e., investigated Table 5). More precisely, none of the need factors was examined among adults in at least half of the studies. Therefore, we displayed the need factor which was examined most frequently in Table 5 (in ten studies out of 29 studies).

### 3.3. Predisposing Characteristics

*Children/adolescents.* In sum, *n* = 10 studies examined predisposing characteristics [22,23,24,25,26,27,28,29,30,31]. Four [23,25,27,31] out of the six studies which examined age found a positive association with dental service use, whereas two studies did not identify such a link [29,30]. While two studies [22,26] found a link between being female and increased dental service use, five studies did not find a significant association between gender and dental service use [23,28,29,30,31]. Other predisposing characteristics were only examined in a few studies. 

*Adults.* In sum, *n* = 31 studies examined predisposing characteristics [32,33,34,35,36,37,38,39,40,41,42,43,44,45,46,47,48,49,50,51,52,53,54,55,56,57,58,59,60,61,62]. With regard to age, five studies [35,42,43,47,59] showed a positive association between age and dental service use, whereas two studies showed a negative association [39,50]. Moreover, 14 studies did not identify a significant association between these factors [33,34,36,37,40,44,46,49,54,55,57,58,60,62]. With regard to gender, while eleven studies found an association between being female and increased dental service use [32,33,35,38,41,43,47,51,54,58,61], four studies found the reverse association [42,48,49,50] and seven studies did not find a significant association [34,36,37,40,44,46,55]. With regard to education, eight studies found a positive association between education and dental service use [33,34,39,44,47,49,53,56], whereas four studies found the reverse association [36,37,41,42] and three studies did not find a significant link [46,54,55]. Other predisposing characteristics were only examined in a few studies. However, it should be noted that most of the studies did not find a significant link between marital status and dental service use [34,36,37,40,44,49,54,55]. Moreover, there was mixed evidence with regard to the association between ethnicity and dental service use.

### 3.4. Enabling Resources

*Children/adolescents.* In sum, *n* = 9 studies examined enabling resources [22,23,24,25,26,27,28,29,30]. Higher family income was associated with increased dental service use in four studies [23,27,29,30], whereas one study did not identify a significant link between these factors [22]. Other enabling resources were only examined in a few studies.

*Adults.* In sum, *n* = 30 studies examined enabling resources. While eight studies found a positive association between income/wealth and dental service use [31,34,39,45,47,57,60,62], three studies found a negative association [42,52,53] and six studies did not identify such a significant association [36,40,43,46,50,54]. Other enabling resources were only examined in a few studies. However, it should be noted that most of the studies found a positive association between social support and dental service use [35,55,57,60] and between usual source of care (i.e., having a dentist an individual usually goes to for dental care) and dental service use [36,37,59,60].

### 3.5. Need Factors

*Children/adolescents.* In sum, *n* = 8 studies examined need factors [22,24,25,26,27,28,30,31]. Oral health problems were consistently associated with increased dental service use in all respective studies [24,25,27,28]. Other need factors were only examined in a few studies.

*Adults.* In sum, *n* = 29 studies examined need factors. Oral health problems were associated with increased dental service use in six studies [36,37,38,44,52,60], whereas four studies did not identify a significant association [37,49,50,55]. Other need factors were only examined in a few studies. However, it should be noted that there was mixed evidence with regard to the association between several other need factors (e.g., missing teeth, general health status, health problems, oral pain, decayed teeth or need of treatment) and dental service use.

### 3.6. Psychosocial Factors/Personality Characteristics

*Children/adolescents.* In sum, only one study explicitly examined the role of psychosocial factors in dental service use [30]. Vingilis et al. [30] showed that psychological distress was not significantly associated with at least one dental visit during the last two years (yes/no). Personality characteristics were not examined.

*Adults*. No studies exist examining the role of psychosocial factors in dental service use among adults. Personality characteristics were not investigated.

### 3.7. Quality Assessment

The quality assessment of included studies is shown in Table 6. In total, 80% to 100% of the criteria were achieved by the studies. Unclear handling of missing data (50% fulfilled), performance of sensitivity analyses (86% fulfilled) and reporting COI/funding (81%) were the categories with the most unmet criteria.

## 4. Discussion

### 4.1. Main Findings

The aim of this systematic review was to provide an overview of observational studies examining the determinants of dental service use based on the Andersen model.

Among children, particularly higher age (predisposing characteristic), higher income (enabling resource) and more oral health problems (need factor) were associated with increased dental service use. Among adults, findings are, in general, less consistent. However, it should be noted that one half of the studies found an association between increased education (predisposing characteristic) and increased dental service.

The determinants of dental service use (stratified by children and adults) will be shortly discussed in the next paragraphs.

### 4.2. Children/Adolescents

It appears plausible that age (predisposing characteric) is positively associated with increased dental service use since the perceived need for dental service use may increase in later childhood. However, the link between age and dental service use should be further investigated since it can be affected by unobserved confounders. Furthermore, enabling resources such as income may be important for access to dental services in certain countries (e.g., United States). For example, there is a poor access to oral health services in Peru [23]. On a different level, similar challenges exist in the United States [29]. Furthermore, need factors such as oral health problems are important for dental service use. This appears very plausible and indicates that such a need for help entails visits to dentists. Thus, they can have checked their symptoms immediately by dentists. Since only one study examined a psychosocial factor, we refrained from discussing these preliminary results to avoid overinterpreting the data.

### 4.3. Adults

There was mainly mixed evidence with regard to the link between several predisposing characteristics (e.g., gender, age, marital status or ethnicity) and dental service use. In contrast, there is some evidence suggesting a link between higher education and increased dental service use. This may be explained by the fact that higher education is associated with higher health literacy [64] which in turn is associated with health promoting behavior [65,66]. There was also mixed and inconclusive evidence with regard to the link between enabling resources and dental service use—even when we only compare the association between income and dental service solely within one country such as the United States [34,37,39,40,45,54,57]. However, it should be noted that social support was associated with increased dental service use. A possible explanation is that relatives or friends may urge the individuals to visit a dentist in case of need. Another explanation is that family members of friends may ease the access to dental services (e.g., transport)—a factor which may become particularly important in late life. Thus, the living situation (e.g., living alone or living with family members) may be of importance and should be further investigated. Unexpectedly, need factors such as oral health, missing teeth, general health status, health problems, oral pain, decayed teeth or need of treatment were not consistently associated with dental service use. One possible explanation is that other factors such as dental anxiety or dental fear [58] may particularly drive dental service use in adulthood. Other studies (not based on the Andersen model) already demonstrated the importance of dental anxiety for dental service use [67].

### 4.4. Differences in Determinants of Dental Service use between Children and Adults

The fact that determinants of dental service use seem to differ between children and adults may be partly explained by the fact that data from countries such as Brazil, Peru or the United States [23,29,30] were used in the studies investigating children. Enabling resources such as income may be of particular importance for dental service use in these countries where there is a poor access to dental services. More generally, it should be emphasized that some degree of the inconsistency in the results may be attributed to the fact that health care systems vary in the countries (e.g., publicly funded healthcare system vs. private healthcare systems).

### 4.5. Comparability of Studies

Regarding comparability of studies, dental service use was often quantified as dental service use (last six months to ever visiting a dentist) and was based on self–reports. This may introduce some recall bias since recall periods up to twelve months have been recommended in previous research [68].

Our systematic review also revealed that most studies were cross–sectional. Only a few longitudinal studies exist. Nevertheless, longitudinal studies are required to gain further insights into the factors leading to dental service use and to deliver consistent estimates [69]. Moreover, studies from very different regions of the world (with different access to dental services (e.g., between emerging and industrialized countries) and different regulations for copayments) were included in our review.

### 4.6. Study Quality

In total, the study quality between the studies only varied slightly. Furthermore, the quality of the studies was relatively high—which may be partly explained by the fact that about one half of the studies have been published since 2017. Some have in common that they did not conducted robustness checks (sensitivity analyses). Robustness checks, however, are required to show the validity and credibility of the results. These checks are also recommended by current guidelines [70]. Furthermore, approximately one half of the studies did not clarify the way missing data were treated. However, this can have various consequences (in terms of biased estimates or marked loss of statistical power [71]). Techniques like full–information maximum likelihood [72] can lead to more reliable results and therefore could be applied in upcoming studies.

### 4.7. Gaps in Knowledge and Guidance for Future Research

This systematic review identified several gaps in knowledge. First, longitudinal studies are required to clarify the determinants of dental service use. Second, studies based on nationally representative samples are needed. Third, psychosocial and personality–related factors should be further examined. Fourth, studies from African countries are required. Fifth, the determinants of preventive dental service use should be further explored. Sixth, the large majority of studies focused on patient–related characteristics. Thus, future studies are required drawing attention to the characteristics related to the dentist and the dentist office. Seventh, dental service use in times of the COVID–19 pandemic should be further explored [73].

### 4.8. Strengths and Limitations

Some strengths and limitations regarding our current systematic review are worth noting. Our current work is the first systematic review regarding the determinants of dental service use drawing on the Andersen model. We conducted a quality assessment. Additionally, two reviewers performed important procedures (study selection, extracting the data and evaluation of study quality). While the restriction to include only peer–reviewed article may assure a high quality of the studies included, this restriction may be accompanied by the exclusion of some existing research (e.g., grey literature). Moreover, due to the language restrictions (i.e., published in English or German language), some studies may not be determined. Moreover, a meta–analysis was not performed due to study heterogeneity. Furthermore, future research is required to specifically focus on gender differences in dental service use.

## 5. Conclusions

Our systematic review revealed that all components (i.e., predisposing characteristics such as age, enabling resources such as income and need factors such as oral health problems) of the Andersen model tend to be associated with dental service use among children, whereas the findings are more mixed among adults. In conclusion, beyond need factors, dental service use also tend to be driven by other factors. This may indicate over—or, more likely—underuse of dental services and could enrich the inequality discussion in dental services research.

## Figures and Tables

**Table 1:** Search strategy (PubMed).

#	Search Term
#1	Dental serv *
#2	Dental visit *
#3	Dental care u *
#4	Oral health serv *
#5	Dentist
#6	#1 OR #2 OR #3 OR #4 OR #5
#7	Andersen model
#8	Andersen’s behavioral model of health serv *
#9	Andersen and Newman behavioral model of health serv *
#10	#7 OR #8 OR #9
#11	#6 AND #10

The asterisk (*) is a truncation symbol. The number sign (#) refers to the search order.

**Table 2:** Study overview and main findings (children and adolescents).

First Author	Country	Assessment of Dental Service Use	Study Type/ Time	Sample	Sample Size; Age; Females in Total Sample	Predisposing Factors	Enabling Factors	Need Factors
Al Agili (2020) [22]	Saudi Arabia	ever visited a dentist (yes/no)	cross-sectional study	third- and eighth-grade children in Jeddah, Saudi Arabia	*n* = 1397 <9 years: 49.8% 9–14 years: 39.4% >14 years: 10.9% 41.0% female children	Multiple logistic regressions showed that parent education (>high school, OR: 2.0 (95% CI: 1.3–3.1), compared to < high school) was associated with an increased likelihood of having ever visited a dentist among third-grade children, whereas sex and nationality were not associated with ever having visited a dentist among third-grade children. Among eighth-grade children, sex (OR: 2.4, 95% CI: 1.4–4.0) was a significant positive predictor, whereas nationality and parental education remained insignificant.	Furthermore, regressions showed that enabling factors (in terms of school type, family monthly income, government financial support and medical insurance) were not associated with the outcome measure among both third- and eighth-grade children.	Regressions showed that carrying experience was associated with an increased likelihood of having ever visited a dentist among both third-grade (OR: 2.8, 95% CI: 1.7–4.7) and eighth-grade children (OR: 2.3, 95% CI: 1.4–3.8).
Azañedo (2017) [23]	Peru	access to an oral health service within the previous six months (yes/no)	cross-sectional study	Survey on Demography and Family Health 2014–2015	*n* = 71,614 0–2: 28.7% 3–5: 27.2% 6–11: 44.1% 49.0% female children	Poisson regression showed that age group (6–12: OR: 3.1, 95% CI: 2.9–3.2) was positively associated with dental services use. Gender was not significantly associated.	Enabling factors: Natural region of residency (jungle: OR: 0.8, 95% CI: 0.8–0.8) was negatively, the type health insurance (private: OR: 1.3, 95% CI: 1.1–1.6) was positively, the quintile of wealth (fifth quintile: OR: 1.6, 95% CI: 1.5–1.7) was positively and caregiver’s educational level (higher: OR: 1.6, 95% CI: 1.5–1.8) was also positively related to dental services use. The area of residence and the caregiver’s language were no significant predictors.	not investigated
Baldani (2011) [24]	Brazil	at least one dental visit in one’s lifetime (yes/no)	cross-sectional study	broad household survey in Paraná, Brazil	*n* = 350 0–6: 52.6% 7–14: 47.4% 51.4% female children	Logistic regression showed that never having had a dental visit was positively associated with only visiting a dentist in case of pain (OR: 4.3, 95% CI: 1.8–10.2), but not significantly with the ownership of one’s family house.	Never having had a dental visit was positively associated with not going to kindergarten or school (OR: 11.2, 95% CI: 5.7–22.1) and not having the health condition regularly monitored by a Family Help Program team (OR: 2.5, 95% CI: 1.3–4.8).	Having reported one’s child oral health problems was negatively related to never having attended a dentist (OR: 0.3, 95% CI: 0.1–0.5).
Baldani (2017) [25]	Brazil	having consulted a dental assistant in one’s lifetime (yes/no)	cross-sectional study	preschool children served by the Family Health Strategy in Paraná, Brazil	*n* = 438 3: 37.7% 4: 34.9% 5: 27.4% 50.7% female children	Poisson regression showed that age group (4: OR: 1.49, 95% CI: 1.0–2.2) was positively related to dental attendance. Household overcrowding was not significant.	Living with both parents was significantly positively associated with an increased probability of dental attendance (OR: 1.5, 95% CI: 1.0–2.2).	An oral impact on the quality of life was a positively related independent variable (OR: 1.6, 95% CI: 1.1–2.2).
Chertok (2018) [26]	United States	at least one dental visit during the last year (yes/no)	cross-sectional study	Youth Risk Behavior Survey (YRBS)	*n* = 5814 ≤14: 3.9% 15: 17.3% 16: 27.3% ≥17: 51.5% 48.3% female children	Logistic regression showed that male gender (OR: 0.9, 95% CI: 0.8–1.0), non-white ethnicity (e.g. Hispanic: OR: 0.4, 95% CI: 0.3–0.6), tobacco use (OR: 0.8, 95% CI: 0.6–0.9), substance abuse (OR: 0.8, 95% CI: 0.7–1.0), not drinking soda (OR: 0.8, 95% CI: 0.7–1.0) and never or rarely wearing a seat belt (OR: 0.5, 95% CI: 0.4, 0.7) were related to decreased odds of dental visits.	Not speaking English well was associated with a decreased likelihood of dental visits (OR: 0.2, 95% CI: 0.1–0.4).	Overweight was associated with decreased odds of dental attendance (OR: 0.7, 95% CI: 0.6–0.9).
Gao (2020) [27]	China	at least one dental visit during the last year (yes/no)	cross-sectional study	National Oral Health Survey	*n* = 40,305 3: 30.7% 4: 34.6% 5: 34.7% 49.8% females	Logistic regression showed that dental attendance was significantly associated with higher age (OR: 1.3, 95% CI: 1.2–1.5), higher parents’ education (OR: 2.3, 95% CI: 2.0–2.7), a high oral health attitude (OR: 1.6, 95% CI: 1.4-1.9) and a high oral health knowledge (OR: 1.4, 95% CI: 1.2–1.5).	Rural location (OR: 0.7, 95% CI: 0.5–0.8) and higher income (OR: 1.4, 95% CI: 1.2–1.7) significantly influenced dental attendance.	Toothache (OR: 9.7, 95% CI: 7.8–12.1) and bad oral health (OR: 3.5, 95% CI: 2.8–4.4) were associated with increased odds, a bad overall health (OR: 0.6, 95% CI: 0.4–0.8) was associated with decreased odds of dental attendance.
Maffioletti (2020) [28]	Brazil	at least one dental visit during the last year (yes/no)	cross-sectional study	12-year old children enrolled in public schools located in a deprived area of the city of Manaus, Brazil	*n* = 358 12-year olds 58.4% female children	According to a Parsimonious model, dental attendance was associated with parents’ sense of coherence (ß = –0.1, *p* < 0.05). The child’s gender remained insignificant.	A higher socioeconomic status was linked to decreased odds of dental attendance (ß = −0.2, *p* < 0.05).	The oral clinical status was significantly related to dental visits (ß = 0.2, *p* < 0.05).
Naavaal (2017) [29]	United States	at least one dental visit during the last six months because of a dental problem (yes/no)	cross-sectional study	National Health Interview Survey	*n* = 2834 2–17 female: not displayed	Logistic regression showed that living in the Midwest was associated with a lower likelihood of dental attendance (OR: 0.8, 95% CI: 0.6–1.0). Age, sex, place of birth and race remained insignificant.	Parents’ college degree (OR: 1.7, 95% CI: 1.1–2.6) and a high family income (e.g. ≥ 300%: OR: 2.6, 95% CI: 1.6–3.4) were related to higher chances of dental attendance.	not investigated
Vingilis (2007) [30]	Canada	at least one dental visit during the last two years (yes/no)	longitudinal study	Canadian National Population Health Survey (NPHS)	*n* = 1493 M = 15.5 (first wave) not displayed	At the second wave, Poisson regression revealed that age, sex, family structure and school or work setting were insignificant.	Income during the first wave (ß = 0.1, *p* < 0.001) and social involvement during the second wave (ß = 0.1, *p* < 0.001) were linked to increased chances of dental attendance.	A better self-rated health (e.g. good: ß = –0.5, *p* < 0.01) and disability (ß = 0.2, *p* < 0.01) during the first wave were associated with decreased chances of dental attendance. Overall health status was not significant.
Xu (2018) [31]	China	at least one dental visit during the last year (yes/no)	cross-sectional study	preschool children from five kindergartens in Beijing, China	*n* = 1425 ≤3: 35.5% ≥4: 64.5% 48.4% females	According to negative binomial regression, being older than three years (OR: 1.5, 95% CI: 1.2–1.8), the better education of one’s parents (e.g. master: OR: 1.4, 95% CI: 1.1–1.7) and Kindergarten attendance with regular source of oral health (OR: 2.2, 95% CI: 1.8–2.8) were related to dental visits. Gender, parents’ oral health knowledge and attitude score and occupation or income remained insignificant.	not investigated	Parental perceived bad oral health status of child (OR: 2.1, 95% CI: 1.6–2.8), decayed or missing teeth (OR: 1.0, 95% CI: 1.0–1.1) and dental pain during the last twelve months (OR: 2.1, 95% CI: 1.7–2.5) were related to increased odds of dental attendance.

**Table 3:** Study overview and main findings (adults).

First Author	Country	Assessment of Dental Service Use	Study Type/Time	Sample	Sample Size; Age; Females in Total Sample	Predisposing Factors	Enabling Factors	Need Factors
Astrom (2013) [32]	Sweden	using dental services at least once a year (yes/no)	longitudinal study (four waves from 1992 to 2007)	recruited among everyone who was born in 1942 and lived in Orebro and Ostergotland, Sweden, in 1992	*n* = 4143 1942 birth cohort 52.2% females	Regression analysis showed that female gender (OR: 1.4, 95% CI: 1.1–1.7) and being married (OR: 1.6, 95% CI: 1.2–2.2) were positively associated with dental services use, while the country of birth was not a significant predictor.	Good quality of dental care (OR: 1.2, 95% CI: 1.0–1.4), dental care as a child (OR: 0.7, 95% CI: 0.5–0.9) and public care (OR: 0.2, 95% CI: 0.2–0.3) were significantly positively related to dental services use, whereas the receive of information during the last visit was not significant.	Missing no teeth (OR: 1.3, 95% CI: 1.0–1.8) was positively and perceived problems (OR: 0.7, 95% CI: 0.6–0.8) was negatively associated with dental attendance.
Born (2006) [33]	Germany	at least one dental visit during the last year (yes/no)	cross–sectional study	Study of Health in Pomerania (SHIP)	*n* = 4310 M = 50.3 SD = 16.4 20–79 50.9% females	Logistic regression revealed that dental attendance was negatively related to a lower education (having completed secondary school: OR: 0.4, 95% CI: 0.2–0.6) and positively related to being female (OR: 1.5, 95% CI: 1.2–1.8). Age was no significant predictor.	Private health insurance (OR: 2.6, 95% CI: 1.5–4.4) and using a bonus booklet (OR: 8.2, 95% CI: 6.3–10.6) led to an increased likelihood of dental attendance.	Not holding regular dental attendance for important (OR: 0.1, 95% CI: 0.1–0.3) was negatively on the one side, being satisfied with one’s teeth appearance (OR: 1.7, 95% CI: 1.1–2.6), still having one’s own teeth (OR: 3.3, 95% CI: 2.2–4.8) and Caries (OR: 1.0, 95% CI; 1.0–1.0) were positively associated with dental visits. In addition, the reason for one’s last dental visit (e.g. prevention: OR: 1.4, 95% CI: 1.1–1.8) was a significant predictor.
Branch (1981) [34]	United States	at least one dental visit during the last year (yes/no)	cross–sectional study	noninstitutionalized elders aged 65 years or older	*n* = 1625 M = 73.2 ≥65 60.0% females	Regression analysis found out that education (ß = 0.1, *p* < 0.05) was significantly associated with dental attendance. Race, age, gender, household composition and marital status were not.	Income (ß = 0.1, *p* < 0.05) and occupation (ß = –0.2, *p* < 0.001) were significant independent variables. Insurance, transportation problems and regular physician visits were insignificant.	Perceived health status, activities of daily living, physical activity performance, ability to climb stairs or walk a half mile and health problems remained insignificant.
Brzoska (2017) [35]	Germany	at least one dental checkup during the last year (yes/no)	cross–sectional study	German Health Update 2009 and German Health Update 2010	*n* = 41,220 age not reported 59.7% females	Regression analysis revealed that migrant status (OR: 0.7, 95% CI: 0.6–0.7), age (OR: 1.0, 95% CI: 1.0–1.0) and female sex (OR: 1.9, 95% CI: 1.8–2.0) were associated with dental visits.	High socioeconomic status (OR: 2.6, 95% CI: 2.4–2.8), private health insurance (OR: 0.8, 95% CI: 0.7–0.8), living in a partnership (OR: 1.6, 95% CI: 1.6–1.7) or in Western Germany (OR: 0.9, 95% CI: 0.8–0.9) or in an urban setting (OR: 0.9, 95% CI: 0.9, 1.0) and strong social support (OR: 1.4, 95% CI: 1.3–1.5) were significantly related to dental visits.	not investigated
Davidson (1997) [36]	United States	at least one dental visit during the last year (yes/no)	cross–sectional study	WHO International Collaborative Study of Oral Health Outcomes (ICS–II)	*n* = 4386 35–44: 52.0% 65–74: 48.0% female: not displayed	Regarding White people in Baltimore, regression showed that nine to eleven education years (OR: 0.4, 95% CI: 0.2–0.8), wearing dentures (OR: 0.5, 95% CI: 0.3–1.0) and being edentulous (OR: 0.1, 95% CI: 0.1–0.2), not being afraid of the visit (OR: 1.7, 95% CI: 1.3–2.2) and motivation to visit (OR: 2.7, 95% CI: 1.7–4.1) were associated with the chances of dental attendance. Age, gender, marital status, general health and other oral health beliefs remained insignificant. Regarding African–Americans, being edentulous (OR: 0.3, 95% CI: 0.1–0.9) and not being afraid of the visit (OR: 1.6, 95% CI: 1.1–2.2) was related to the odds of dental attendance. Age, gender, education, marital status, general health, wearing dentures and other health beliefs remained insignificant.	Among White people, the presence of a usual source of care was associated with an increased likelihood of a dental visit (OR: 30.1, 95% CI: 15.4–58.8). Income and dental benefits remained insignificant. Among African–Americans, the presence of a usual source of care was associated with increased odds of a dental visit (OR: 6.7, 95% CI: 2.9–15.5). Income and dental benefits remained insignificant.	For White people, oral pain was associated with higher chances of dental attendance (OR: 1.8, 95% CI: 1.3–2.7). Oral symptoms were not.For African Americans, oral pain was related to increased odds of dental attendance (OR: 1.7, 95% CI: 1.1–2.2). Oral symptoms remained insignificant.
Davidson (1999) [37]	United States	at least one dental visit during the last year (yes/no)	cross–sectional study	WHO International Collaborative Study of Oral Health Outcomes (ICS–II)	*n* = 4412 35–44: 60.5% 65–74: 39.5% 53.6% females	Regarding regression analysis for Baltimore, being White (OR: 2.0, 95% CI: 1.4–2.9), nine to eleven education years (OR: 0.5, 95% CI: 0.3–0.9), being edentulous (OR: 0.1, 95% CI: 0.1–0.2), not being afraid of dental visits (OR: 1.6, 95% CI: 1.3–2.0) and a motivation to visit (OR: 2.0, 95% CI: 1.5–2.8) were related to dental attendance. Age, gender, marital status, general health, dentures, thinking that oral health is important and having a dentist available remained insignificant.	With regard to Baltimore, having a usual source of care (OR: 16.9, 95% CI: 10.0–28.6) was associated with increased odds of dental attendance. Income and dental visits remained insignificant.	Referring to Baltimore, oral pain was related to increased odds of dental attendance (OR: 1.6, 95% CI: 1.2–2.3). The number of oral symptoms was not.
Ekanayake (2002) [38]	Sri Lanka	at least one dental visit during the last two years (yes/no)	cross–sectional study	employees of public sector institutions situated in the city of Kandy	*n* = 210 21–34: 27.1% 35–45: 47.6% >45: 25.3% 50.5% females	Logistic regression showed that female gender was associated with an increased likelihood of dental attendance (OR: 2.5, 95% CI: 1.4–4.7)	not investigated	Dental pain within the last six months was related to increased odds of a dental visit (OR: 2.0, 95% CI: 1.0–4.0).
Evashwick (1984) [39]	United States	at least one dental visit during the last 15 months (yes/no)	longitudinal study (wave 1: 1974, wave 2: 1976)	Massachusetts Health Care Panel Study	*n* = 1317 65–69: 36.2% 70–74: 27.4% 75–89: 19.7% ≥80: 16.7% 61.7% females	Education and preventive visits at one’s physician were associated with more dental visits, higher age with less dental visits. Widowed use and race remained insignificant, according to multiple regression analysis.	Higher income and a white collar job were significantly associated with an increased likelihood of dental attendance. Having Medicaid, a doctor or transportation problems were not predictive.	A bad health state was linked to decreased odds of dental attendance. Problems with physical activities, walking stairs or half a mile and a poor function status or physical condition were insignificant.
Finlayson (2010) [40]	United States	at least one dental visit during the last year (yes/no)	cross–sectional study	UC Davis Immigration to California: Agricultural Safety and Acculturation (MICASA) study	*n* = 326 M = 36.7 SD = 9.0 20–61 67.5% females	Regarding Generalized estimating equation logit regression, asking for the dentist’s advice was associated with a higher likelihood of dental attendance (OR: 4.6, 95% CI: 2.3–9.5). Age, gender, being married, days worked farming and fair or poor health were insignificant.	Having a regular source of dental care was related to higher chances of having had a dental visit (OR: 4.8, 95% CI: 2.5–9.4). Acculturation, education, income, household size and dental insurance status were not significant.	Self–reported symptoms were associated with decreased odds of dental attendance (OR: 0.9, 95% CI: 0.8–0.9). Untreated decay, gum bleeding on probing and subjective need remained insignificant.
Fonseca (2020) [41]	Brazil	at least one dental visit during the last year (yes/no)	cross–sectional study	representative sample of adults living in the State of São Paulo	*n* = 5709 35–39: 51.2% 40–45: 48.8% 68.0% females	Logistic regression showed that male gender (OR: 0.9, 95% CI: 0.7–1.0) and 10 or more education years (OR: 0.5, 95% CI: 0.5–0.6) were associated with decreased, and that non–white skin color (OR: 1.3, 95% CI: 1.2–1.5) and toothache (OR: 1.6, 95% CI: 1.4–1.8) were associated with increased odds of dental attendance.	Lower household income was related to increased odds of a dental visit (OR: 2.4, 95% CI: 2.1–2.7).	Endodontic treatment was significantly linked to dental attendance (OR: 1.4, 95% CI: 1.1–1.9).
Herkrath (2018) [42]	Brazil	at least one dental visit during the last year (yes/no)	cross–sectional study	Brazilian National Health Survey (NHS)	*n* = 27,017 18–21: 8.5% 22–34: 29.2% 35–44: 19.8% 45–64: 30.0% ≥65: 12.5% 55.0% females	Multilevel logistic regression revealed that higher age (e.g. 65+: OR: 3.2, 95% CI: 2.7–3.8), male gender (OR: 1.5, 95% CI: 1.4–1.6), brown race (both: OR: 1.1, 95% CI: 1.0–1.2), less years of schooling (e.g. 0–4: OR: 2.1, 95% CI: 1.9–2.4) and a low social network (OR: 1.6, 95% CI: 1.4–1.8) were related to an increased likelihood of dental visits.	Lower income (OR: 1.6, 95% CI: 1.4–1.8) and having no health insurance (OR: 1.8, 95% CI: 1.7–1.9) were significantly associated with higher odds of dental attendance.	A poor perceived dental health (OR: 1.8, 95% CI: 1.5–2.1) and missing all teeth (OR: 2.9, 95% CI: 2.4–3.3) were positively related to dental visits, while eating difficulties due to an oral problem (OR: 0.9, 95% CI: 0.8–1.0) and missing one or more teeth (OR: 0.8, 95% CI: 0.7–0.8) were negatively related.
Herkrath (2020) [43]	Brazil	ever visited a dentist (yes/no)	cross–sectional study	Brazilian National Health Survey (NHS)	*n* = 60,202 M = 42.9 95% CI: 42.9–43.0 52.9% females	Regression analysis showed that being male and being younger were related to decreased chances of dental attendance.	Living in urban areas, higher enabling financing and public health center registration were related to increased odds of dental attendance.	Higher perceived needs were associated with higher chances of dental attendance.
Jang (2019) [44]	United States	total count of visits to a dentist during the last year	cross–sectional study	representative sample of Korean immigrants from five cities	*n* = 2128 M = 73.4 SD = 8.0 60–100 66.8% females	Regarding the Poisson regression, having at least a high school degree was associated with a higher likelihood of dental attendance (OR: 1.1, 95% CI: 1.0–1.2). Age, gender, marital status and region remained insignificant.	Dental insurance coverage (OR: 1.4, 95% CI: 1.2–1.5) and the presence of a family network (OR: 1.0, 95% CI: 1.0–1.0) were significantly associated with a higher likelihood of dental visits. Acculturation and the length of stay in the United States were not significant.	A problem with teeth or gums was related to increased odds of dental attendance (OR: 1.1, 95% CI: 1.0–1.2). A fair or poor rating of one’s oral health was not significant.
Kiyak (1987) [45]	United States	any use of dental services during the last three years (yes/no)	cross–sectional study	low–income and middle–income elderly, recruited from medical centers with reduced service fees	*n* = 258 M = 73.6 71.9% females	Multiple regression revealed that one’s importance (b = –0.3, ß < 0.01) and one’s gender (b = 0.1, ß < 0.05) were significantly related to dental attendance.	One’s beliefs (b = –0.1, ß < 0.05), one’s income (b = 0.1, ß < 0.05) and one’s information (b = –0.0, ß < 0.05) were significantly associated with dental attendance.	The number of teeth (b = –0.0, ß < 0.01), one’s perceived need (b = –0.3, ß < 0.01) and wearing a denture (b = 0.2, ß < 0.05) were associated with increased or decreased odds of dental attendance.
Lee (2020) [46]	South Korea	any use of dental services during the last three years (yes/no)	cross–sectional study	nationwide sample of homeless people	*n* = 2032 <50: 26.9% ≥50: 73.1% 19.6% females	According to Poisson regression, drinking was associated with decreased odds of dental attendance (OR: 0.8, 95% CI: 0.7–1.0). Age, sex, education, duration of homelessness and smoking remained insignificant.	Shelter housing (OR: 1.6, 95% CI: 1.1–2.3) and not being employed (OR: 0.8, 95% CI: 0.7–1.0) were related to dental attendance. Income was not significant.	Subjective health and having a medical disease were not significant.
Limpuangthip (2019) [47]	Thailand	any use of public dental services during the last five years (yes/no)	cross–sectional study	randomly selected people aged 50 and above	*n* = 38,695 60–69: 55.7% 70–79: 30.7% ≥80: 13.6% 55.7% females	Binary logistic regression stated that higher age (e.g. 80+: OR: 2.0, 95% CI: 1.8–2.1), female gender (OR: 1.3, 95% CI: 1.2–1.3), a higher education (e.g. at least tertiary: OR: 1.3, 95% CI: 1.1–1.5), higher household possession (e.g. fourth quartile: OR: 2.2, 95% CI: 2.1–2.4) and dependency status (e.g. low dependency: OR: 1.3, 95% CI: 1.2–1.3) were associated with an increased likelihood of dental attendance.	Working in agricultural and related sectors (OR: 0.7, 95% CI: 0.6–0.8), health–promoting behavior (OR: 1.4, 95% CI: 1.2–1.7), alcohol drinking or smoking (OR: 0.8, 95% CI: 0.8–0.9), public healthcare service utilization for vaccination (OR: 1.2, 95% CI: 1.1–1.2) or recent illness (OR: 1.2, 95% CI: 1.1–1.2), treatment by health personnel for recent falling accident (OR: 0.8, 95% CI: 0.8–0.8), being visited by a village health volunteer (OR: 0.8, 95% CI: 0.7–0.8) and participation in a club for the elderly (OR: 1.0, 95% CI: 0.9–1.0) were related to dental attendance. Information awareness was not.	not investigated
Lo (1998) [48]	Hong Kong (China)	at least one dental visit during the last year (yes/no)	longitudinal study (two waves from 1991 to 1992)	random sample of 35– to 44–year–olds from two districts in Hong Kong	*n* = 322 35– to 44–year–olds female: not displayed	Regarding logistic regression, sex (ß = 1.0, *p* < 0.01) was associated with an increased likelihood of having made a dental visit.	Having a dentist as health counselor was associated with dental attendance (ß = 1.5, *p* < 0.01). Being a regular user and toothbrushing remained insignificant.	Need for fillings (ß = 0.9, *p* < 0.05), the number of filled (ß = 0.1, *p* < 0.01) teeth were associated with different odds of dental attendance. The number of decayed teeth was not significant.
McKernan (2018) [49]	United States	at least one dental visit since being enrolled in the study (yes/no)	cross–sectional study	adults enrolled in the Iowa Dental Wellness Plan	*n* = 1258 M = 45.2 SD = 12.4 19–64 40.3% females	Referring to a logistic regression model, female sex (OR: 0.7, 95% CI: 0.5–0.9), chronic physical conditions (OR: 1.4, 95% CI: 1.1–1.9), a high school degree (OR: 0.7, 95% CI: 0.5–0.9) and being edentulous (OR: 0.5, 95% CI: 0.2–0.8) were related to dental attendance. Age, marital status and ethnicity were not significant.	Worry about transportation costs (OR: 0.8, 95% CI: 0.7–0.9) and using public transport systems or walking (OR: 0.6, 95% CI: 0.4–0.9) were related to a decreased likelihood of dental attendance. Urban–rural character, the distance to the nearest dentist and unmet transportation needs were insignificant.	Dental problems interfere with regular activities was insignificant.
Muirhead (2009) [50]	Canada	at least one dental visit during the last year (yes/no)	cross–sectional study	working poor people aged between 18and 64 years from ten Canadian provinces	*n* = 1049 18–24: 14.6% 25–34: 19.7% 35–44: 22.4% 45–54: 22.7% 55–64: 20.6% 41.3% females	With regard to the logistic regression analysis, being male (OR: 1.6, 95% CI: 1.2–2.3) and being 25 to 34 years old (OR: 2.0, 95% CI: 1.1–3.7) was related to dental attendance. Other age groups and lone parent status were not.	Out–of–pocket dental payment (OR: 2.6, 95% CI: 1.6–3.3), competing needs (OR: 0.5, 95% CI: 0.3–0.9) and a history of welfare receipt (OR: 1.7, 95% CI: 1.1–2.6) were significantly associated with dental visits. Income was not.	Being without a functional dentition (OR: 4.2, 95% CI: 2.4–7.4) and perceived need for dental treatment (OR: 2.8, 95% CI: 2.0–3.9) were related to dental attendance. Oral health impact on sleep was not.
Nasir (2009) [51]	Sudan	at least one dental visit during the last two years (yes/no)	cross–sectional study	recruited from a hospital and a university	*n* = 1262 ≤29: 47.6% >30: 52.4% 64.8% females	Regression analysis showed being female (OR: 2.1, 95% CI: 1.4–3.2) were related to increased odds of dental attendance. Travelling outside or inside Sudan was not.	High knowledge of HIV transmission (OR: 0.5, 95% CI: 0.3–0.7) and high experience of HIV (OR: 0.8, 95% CI: 0.5–1.3) were associated with lower odds of dental attendance. Perceived personal risk and attitudes towards people infected with HIV were not.	Filled teeth (OR: 14.9, 95% CI: 3.1–72.1), good teeth condition (OR: 0.5, 95% CI: 0.3–0.8) and good health condition (OR: 0.9, 95% CI: 0.5–1.5) were significantly linked to dental attendance.
Pinto Rda (2014) [52]	Brazil	use of public healthcare services (yes/no)	cross–sectional study	SB Minas Gerais Project	*n* = 1101 35–39: 52.7% 40–44: 47.3% 65.8% females	Being dark–skinned or black (OR: 2.4, 95% CI: 1.3–4.5) and living with more than four people in a household (OR: 2.0, 95% CI: 1.4–2.9) was related to higher odds of dental attendance, according to regression analysis.	A smaller income (e.g. up to 750$: OR: 3.9, 95% CI: 1.8–9.5) and a smaller town size (OR: 3.0, 95% CI: 1.9–4.6) were linked to a higher likelihood of having had a dental visit.	Teeth needing treatment was positively associated with the likelihood of dental attendance (OR: 1.1, 95% CI: 1.0–1.2).
Rebelo Vieira (2019) [53]	Brazil	ever visited a dentist (yes/no)	cross–sectional study	Brazilian Oral Health Survey (SB Brazil Project)	*n* = 7265 35–39: 52.5% 40–44: 47.5% 69.9% females	Multilevel logistic regression showed that high longevity (OR: 0.3, 95% CI: 0.1–1.0), female sex (OR: 0.7, 95% CI: 0.5–0.8), brown skin color (OR: 0.6, 95% CI: 0.4–0.7) and less years of schooling (e.g. 5–8: OR: 1.6, 95% CI: 1.1–2.2) were related to dental non–attendance.	Lower income (e.g. ≤500R$ per month: OR: 4.9, 95% CI: 3.0–8.0) was associated with increased chances of non–attendance.	Perceived dental treatment (OR: 0.4, 95% CI: 0.3–0.6) and one or more decayed teeth (OR: 1.4, 95% CI: 1.1–1.8) were related to dental attendance.
Reisine (1987) [54]	United States	number of dental visits during the last two years	cross–sectional study	university employees	*n* = 287 M = 28.0 61.3% females	According to stepwise regression, being male was associated with increased numbers of dental visits (ß = –0.3, *p* < 0.05). Age, education, marital status, brushing frequency, father’s education, attitude and number of children remained insignificant.	Residence, convenience, transportation and income were not significant.	Decay (ß = –0.1, *p* < 0.05) and missing or filled teeth (ß = 0.2, *p* < 0.05) were associated with dental attendance. Fluoride, the reason for the visit and periodontal pocket measures were not.
Serna (2020) [55]	United States	at least one dental visit during the last year (yes/no)	cross–sectional study	HIV Risk Reduction among Hispanic Migrant Workers in South Florida	*n* = 278 18–49: 71.9% ≥50: 28.1% 45.7% females	A logistic regression model revealed that trying to prevent tooth decay (OR: 2.5, 95% CI: 1.1–5.7) and brushing teeth once a day (OR: 3.9, 95% CI: 1.6–9.4) were associated with an increased likelihood of dental attendance. Age, sex, country of origin, formal education, relationship status, religious beliefs and use of dental floss were not.	Employment status, medical insurance, place of medical care, social support and acculturation remained insignificant.	A good oral health condition was associated with increased odds of dental visits (OR: 3.9, 95% CI: 1.9–7.9). Need of treatment and oral health problems were not.
Silva (2013) [56]	Brazil	at least one dental visit during the last three years (yes/no)	cross–sectional study	users of Family Health Units in the urban area of Pelotas, Brazil	*n* = 438 60–69: 57.4% 70–79: 31.6% ≥80: 11.0% 68.3% females	Poisson regression explored that lower education was associated with lower odds of dental attendance (e.g. < 4 years: OR: 1.4, 95% CI: 1.0–2.0).	Being a former alcohol consumer was associated with higher chances of dental attendance (OR: 1.3, 95% CI: 1.1–1.6).	Having no teeth was related to higher odds of dental attendance (OR: 1.7, 95% CI: 1.3–2.3).
Stapleton (2016) [57]	United States	at least one dental visit during the last year (yes/no)	cross–sectional study	Indiana Black Men’s Health Study	*n* = 1444 18–34: 38.3% 35–44: 18.9% 45–64: 35.1% ≥65: 7.7% 0.0% females	According to multilevel regression, being married (OR: 1.4, 95% CI: 1.1–1.6) was associated with increased chances of dental attendance. Age remained insignificant.	Being a college graduate (OR: 1.8, 95% CI: 1.2–2.8) or employed (OR: 0.7, 95% CI: 0.5–1.0), a higher income (e.g. > $35,000: OR: 1.9, 95% CI: 1.1–3.2), health insurance (OR: 1.7, 95% CI: 1.2–2.3) and high social support (OR: 1.9, 95% CI: 1.3–2.8) were related to dental visits. Smoking and the place of sick care were not.	Three or more fruit servings per day were related to an increased likelihood of dental attendance (OR: 1.8, 95% CI: 1.2–2.8). Self–rated health status, poor mental health days and vegetable servings remained insignificant.
Suominen (2017) [58]	Finland	at least one dental visit during the last year (yes/no)	cross–sectional study	National Health 2000 and 2011 Surveys	*n* = 12,759 aged 30 and older not displayed	In 2011, regarding logistic regression, being female was associated with increased odds of dental visits (OR: 1.2, 95% CI: 1.0–1.4). Age was not significant.	The presence of waiting lists (OR: 1.2, 95% CI: 1.0–1.5) or high costs (OR: 0.5, 95% CI: 0.4–0.8), as a barrier to care, regular check–ups (OR: 3.9, 95% CI: 3.2–4.7), dental fear (OR: 1.1, 95% CI: 1.0–1.3) and being recalled (OR: 1.6, 95% CI: 1.3–2.0) were associated with dental attendance. Poor connection as a barrier to care was not.	Perceived need for care was related to decreased odds of dental attendance (OR: 0.6, 95% CI: 0.5–0.7). Self–rated oral health and wearing removable dentures were not.
Tennstedt (1994) [59]	United States	number of dental visits during the last year	cross–sectional study	community–dwelling, noninstitutionalized elders aged 70 and older, living within the six New England states in the United States	*n* = 3668 M = 77.5 SD = 5.5 70–96 57.0% females	Ordinal logistic regression found out that higher age was associated with a lower number of dental visits (OR: 0.8, *p* < 0.01).	Dental hygiene practices (OR: 1.5, *p* < 0.001), higher education (OR: 1.2, *p* < 0.01) and the presence of a usual source of care (OR: 45.9, *p* < 0.001) were related to dental visits.	Perceived need for care (OR: 0.7, *p* < 0.05), the number of caries (OR: 0.9, *p* < 0.05) and the number of filled teeth (OR: 1.5, *p* < 0.001) was linked to dental attendance.
Varenne (2006) [60]	Burkina Faso	at least one dental visit during the last year (yes/no)	cross–sectional study	people who had an oral problem from four areas representative of different stages of urbanization of Ouagadougou, Burkina Faso	*n* = 809 15–24: 8.8% 25–34: 22.8% 35–44: 34.1% 45–54: 17.3% ≥55: 17.0% 67.4% females	According to logistic regression, being 25 to 34 years old (OR: 2.7, 95% CI: 1.5–4.7), being Christian (OR: 1.8, 95% CI: 1.3–2.6), higher material living conditions of one’s household (e.g. high: OR: 3.4, 95% CI: 2.1–5.4), agreeing that oral diseases are as important as other health problems (OR: 2.1, 95% CI: 1.2–3.6) and disagreeing that going to the dentist is synonymous with pain (OR: 0.5, 95% CI: 0.3–0.7) were related to dental attendance.	Active participation in one’s social network (OR: 1.8, 95% CI: 1.1–3.0) and using a moped or vehicle (OR: 2.2, 95% CI: 1.4–3.2) were associated with an increased likelihood of dental visits.	Oral problem causing limitation or stopping any of usual activities were related to increased odds of dental visits (OR: 3.4, 95% CI: 2.4–4.9).
Xu (2020) [61]	China	at least one dental visit during the last year (yes/no)	cross–sectional study	National Oral Health Survey	*n* = 7206 35–44: 50.9% 65–74: 49.1% 48.9% females	Poisson regression showed that among 35–44–years old people, being female (OR: 1.2, 95% CI: 1.0–1.3) and having a high knowledge about oral health (OR: 1.3, 95% CI: 1.1–1.6) were related to an increased likelihood of dental attendance. Education was not. Regarding 65–74 years old people, being female (OR: 1.3, 95% CI: 1.1–1.6) and having a high education (OR: 1.4, 95% CI: 1.1–1.7) were linked to increased odds of dental attendance. Oral health knowledge remained insignificant.	Location, income, public medical insurance coverage and private medical insurance were not significant among the 35–44–years old. Concerning 65–74 years old, higher income (e.g. third tercile: OR: 1.5, 95% CI: 1.2–2.0) and some kinds of public health insurance (e.g. UEBMI: OR: 1.7, 95% CI: 1.3–2.2) were associated with higher odds of dental visits.	Among 35–44 years old people, worse perceived oral health status (OR: 2.5, 95% CI: 2.0–3.2) and a worse carious status (OR: 1.5, 95% CI: 1.1–2.1) were related to higher chances of dental attendance. Among 65–74 years old people, poor perceived oral health (OR: 1.5, 95% CI: 1.2–2.0) was associated with a higher probability of dental attendance, carious status remained insignificant.
Zlotnick (2014) [62]	Israel	utilization of primary dental care (yes/no)	cross–sectional study	nationwide sample	*n* = 7068 2000 sample: 18–24: 7.9% 25–34: 21.4% 35–44: 19.4% 45–54: 18.8% 55–64: 14.5% ≥65: 18.0% 2010 sample: 18–24: 6.0% 25–34: 15.8% 35–44: 26.0% 45–54: 21.2% 55–64: 16.7% ≥65: 14.2% 2000 sample: 54.8% females 2010 sample: 53.2% females	According to logistic regression, in 2010, regarding Israeli–Jews, being born in Israel was associated with higher odds of dental attendance (OR: 1.5, 95% CI: 1.2–1.8). Among Israeli–Arabs, being older than 65 was related to a higher chance of dental attendance (OR: 0.5, 95% CI: 0.2–1.0). Age remained insignificant.	Among Israeli–Jews, having visited high school (OR: 1.6, 95% CI: 1.2–2.1), being employed (OR: 1.3, 95% CI: 1.1–1.6) having an over average income (OR: 1.9, 95% CI: 1.5–2.3) and flosses (OR: 1.8, 95% CI: 1.4–2.2) were associated with a higher probability of dental attendance. With regard to Israeli–Arabs, having visited high school (OR: 1.6, 95% CI: 1.2–2.2), an over average income (OR: 1.6, 95% CI: 1.3–2.1) and flosses (OR: 2.2, 95% CI: 1.5–3.1) were related to a higher likelihood of dental attendance.	Pain (OR: 0.5, 95% CI: 0.0–0.1), a normal BMI (OR: 1.3, 95% CI: 1.1–1.6) and being a smoker (OR: 0.7, 95% CI: 0.5–0.9) were significantly associated with dental attendance among Israeli–Jews. Among Israeli–Arabs, pain (OR: 0.4, 95% CI: 0.2–0.5) was related to lower chances of dental attendance.

**Table 4:** Key findings (children/adolescents).

Factors	Number of Studies	Positive Relationship	Negative Relationship	No Relationship
Predisposing characteristics	10			
Age	6	4	0	2
Sex (female, ref.: male)	7	2	0	5
Enabling resources	9			
Family income	5	4	0	1
Need factors	8			
Oral health problem	4	4	0	0

**Table 5:** Key findings (adults).

Factors	Number of Studies	Positive Relationship	Negative Relationship	No Relationship
Predisposing characteristics	31			
Age	21	5	2	14
Sex (female, ref.: male)	24	11	6	7
Education	16	8	4	3
Enabling resources	30			
Income or wealth	17	8	3	6
Need factors	29			
Oral health problems	10	6	0	4

**Table 6:** Quality assessment.

First Author (Year)	Study Objective	Inclusion and Exclusion Criteria	Dental Visits Description	Data Source	Missing Data	Statistics	Consideration of Confounders	Sensitivity Analysis	Sample Size (Subgroup)	Demographics	Results Discussed with Respect to Other Studies	Results Discussed Regarding Generalizability	Limitations	Conclusion Supported by Data	Conflict of Interest/Funders
Al Agili (2020) [22]	X	X	X	X	X	X	X	X	X	X	X	X	X	X	X
Astrom (2013) [32]	X	X	X	X	X	X	X	X	X	X	X	X	X	X	X
Azañedo (2017) [23]	X	X	X	X		X	X	X	X	X	X	X	X	X	X
Baldani (2011) [24]	X	X	X	X		X	X	X	X	X	X	X	X	X	X
Baldani (2017) [25]	X	X	X	X	X	X	X	X	X	X	X	X	X	X	X
Born (2006) [33]	X	X	X	X		X	X	X	X	X	X	X		X	
Branch (1981) [34]	X	X	X	X		X	X	X	X	X	X	X	X	X	X
Brzoska (2017) [35]	X	X	X	X		X	X	X	X	X	X	X	X	X	X
Chertok (2018) [26]	X	X	X	X		X	X	X	X	X	X	X	X	X	X
Davidson (1997) [36]	X	X	X	X		X	X	X	X	X	X	X	X	X	X
Davidson (1999) [37]	X	X	X	X	X	X	X	X	X	X	X	X	X	X	X
Ekanayake (2002) [38]	X	X	X	X		X	X	X	X	X	X	X	X	X	
Evashwick (1984) [39]	X	X	X	X	X	X	X	X	X	X	X	X	X	X	X
Finlayson (2010) [40]	X	X	X	X	X	X	X	X	X	X	X	X	X	X	X
Fonseca (2020) [41]	X	X	X	X		X	X	X	X	X	X	X	X	X	
Gao (2020) [27]	X	X	X	X	X	X	X	X	X	X	X	X	X	X	X
Herkrath (2018) [42]	X	X	X	X		X	X	X	X	X	X	X	X	X	X
Herkrath (2020) [43]	X	X	X	X	X	X	X	X	X	X	X	X	X	X	X
Jang (2019) [44]	X	X	X	X	X	X	X		X	X	X	X	X	X	X
Jönsson (2020) [63]	X	X	X	X	X	X	X	X	X	X	X	X	X	X	X
Kiyak (1987) [45]	X	X	X	X		X	X	X	X	X	X	X	X	X	X
Lee (2020) [46]	X	X	X	X	X	X	X	X	X	X	X	X	X	X	X
Limpuangthip (2019) [47]	X	X	X	X		X	X		X	X	X	X	X	X	X
Lo (1998) [48]	X	X	X	X	X	X	X	X	X	X	X	X	X	X	X
Maffioletti (2020) [28]	X	X	X	X		X	X	X	X	X	X	X	X	X	X
McKernan (2018) [49]	X	X	X	X		X	X		X	X	X	X	X	X	X
Muirhead (2009) [50]	X	X	X	X		X	X		X	X	X	X	X	X	
Naavaal (2017) [29]	X	X	X	X		X	X	X	X	X	X	X	X	X	
Nasir (2009) [51]	X	X	X	X		X	X		X	X	X	X	X	X	X
Pinto Rda (2014) [52]	X	X	X	X		X	X	X	X	X	X	X	X	X	X
Rebelo Vieira (2019) [53]	X	X	X	X	X	X	X	X	X	X	X	X	X	X	X
Reisine (1987) [54]	X	X	X	X		X	X	X	X	X	X	X	X	X	
Serna (2020) [55]	X	X	X	X		X	X	X	X	X	X	X	X	X	
Silva (2013) [56]	X	X	X	X		X	X		X	X	X	X	X	X	X
Stapleton (2016) [57]	X	X	X	X	X	X	X	X	X	X	X	X	X	X	X
Suominen (2017) [58]	X	X	X	X		X	X	X	X	X	X	X	X	X	X
Tennstedt (1994) [59]	X	X	X	X	X	X	X	X	X	X	X	X	X	X	
Varenne (2006) [60]	X	X	X	X		X	X	X	X	X	X	X	X	X	X
Vingilis (2007) [30]	X	X	X	X	X	X	X	X	X	X	X	X	X	X	X
Xu (2018) [31]	X	X	X	X		X	X	X	X	X	X	X	X	X	X
Xu (2020) [61]	X	X	X	X	X	X	X	X	X	X	X	X	X	X	X
Zlotnick (2014) [62]	X	X	X	X		X	X	X	X	X	X	X	X	X	X
	100	100	100	100	49.5	100	100	85.7	100	100	100	100	97.6	100	81.0

## Data Availability

Not applicable.

## Supplementary Materials

The following are available online at https://www.mdpi.com/1660-4601/18/5/2491/s1, Table S1: Key findings (children/adolescents)—extended, Table S2: Key findings (adults)—extended.
