# Supplementary material for: Factors Associated with Dental Service Use Based on the Andersen Model: A Systematic Review
Source: Int J Environ Res Public Health. 2021 Mar 3;18(5):2491. doi: 10.3390/ijerph18052491 (PMC7967618; doi:10.3390/ijerph18052491)
Supplement: Supplementary file 1 [file ijerph-18-02491-s001.pdf]

International Journal of
Environmental Research and Public Health

MDPI

Table S1. Key findings (children/adolescents) - extended 1

	Number of studies	Positive relationship	Negative relationship	No relationship
**Predisposing characteristics**	10			
Age	6	4	0	2
Parent's education	3	3	0	0
Sex (female, ref.: male)	7	2	0	5
Nationality or place of birth (ref.: foreign)	2	0	0	2
Family income or wealth	2	0	0	2
High oral health attitude	3	2	0	1
Household overcrowding	1	0	0	1
Ethnicity (white, ref.: non-white)	2	0	1	1
Tobacco use†	1	0	1	0
Substance abuse	1	0	1	0
Drinking soda	1	0	1	0
Never or rarely wearing a seatbelt	1	0	1	0
High oral health knowledge	2	1	0	1
Parent's sense of coherence	1	0	1	0
Residence (natural or rural, ref.: urban)	1	0	1	0
Family structure (ref.: living with two parents in a household)	1	0	0	1
School setting (ref.: public)	1	0	0	1
Work setting (ref.: not working)	2	0	0	2
Regular source of oral health	1	1	0	0
**Enabling resources**	9			
Family income	5	4	0	1
School type (ref.: public)	1	0	0	1
Government financial support or family help program	2	1	0	1
Medical insurance	2	1	0	1

Residence (natural or rural, ref.: urban)	3	0	2	1
Caregiver's education	2	2	0	0
Caregiver speaking local language well	2	1	0	1
Going to kindergarten or school	1	1	0	0
Living with both parents	1	1	0	0
Socioeconomic status	1	1	0	0
Social involvement	1	1	0	0
**Need factors**	8			
Carrying experience	1	1	0	0
Oral health	1	1	0	0
Oral health problem	4	4	0	0
Overweight	1	0	1	0
Disability	1	0	1	0
Overall health	1	0	0	1
Overall health problem	1	0	1	0
Self-rated health	1	0	1	0
Decayed or missing teeth	1	1	0	0
Dental pain	1	1	0	0
**Psychosocial factors**	1			
Psychological distress	1	0	0	1

Table S2. Key findings (adults) - extended

	**Number of studies**	**Positive relationship**	**Negative relationship**	**No relationship**
**Predisposing characteristics**	31			
Age	21	5	2	14
Sex (female, ref.: male)	24	11	4	7
Marital status (living in a partnership, ref.: single)	12	3	0	8
Ethnicity (white, ref.: non-white)	11	3	4	4
Education	16	8	4	3
Household size	3	1	0	2
Wearing dentures	1	0	1	2
Being edentulous	3	0	3	0
Not being afraid of the visit	2	2	0	0

Motivation to visit	4	4	0	0
Oral health beliefs	3	0	0	3
General health	4	0	1	3
Having a dentist available	1	0	0	1
Asking for the dentist's advice	1	1	0	0
Days worked farming	1	0	0	1
Toothache	1	1	0	0
Social network	1	0	1	0
Being religious	2	1	0	1
Importance	1	0	1	0
Drinking	1	0	1	0
Smoking	1	0	0	1
Income or wealth	2	2	0	0
Dependency	1	0	1	0
Travelling	1	0	0	1
Toothbrushing	2	1	0	1
Number of children	1	0	0	1
Former drinking	1	1	0	0
Use of dental floss	1	0	0	1
Religious beliefs (ref.: no)	1	0	0	1
Thinking that going to the dentist is synonymous with pain	1	0	1	0
**Enabling resources**	30			
Income or wealth	17	8	3	6
Good quality of dental care	1	1	0	0
Dental care as a child	1	0	1	0
Public care	7	4	1	2
Health or health care knowledge	3	0	2	1
Health or dental insurance	8	3	2	3
Dental benefits	3	1	0	2
Occupation (white collar, ref.: blue collar)	3	3	0	0
Transportation problems	4	0	1	3
Regular medical visits	3	1	0	2
Socioeconomic status	1	1	0	0
Living place (former socialist regime, ref.: capitalist regime)	1	1	0	0

Living place (rural, ref.: urban)	6	2	1	3
Social support	5	4	0	1
Usual source of care	6	5	0	1
Dental visits	1	0	0	1
Acculturation	3	0	0	3
Education	4	3	0	1
Household size	1	0	0	1
Length of stay in one's country	1	0	0	1
Health beliefs (ref.: not motivated to or afraid of visit)	2	1	1	0
Unemployment	4	2	1	1
Alcohol drinking or smoking	1	0	1	0
Recent illness	1	1	0	0
Having a caregiver due to a falling accident	1	0	1	0
Being a village health volunteer	1	0	1	0
Participation in a club for the elderly	1	0	1	0
Dental hygiene practice	2	1	0	1
Use of public transport systems or walking (vs. motorized vehicle)	2	0	2	0
Poor connection or long distance to a dentist	2	0	0	2
Out-of-pocket dental payment	1	1	0	0
Competing needs	1	0	1	0
HIV knowledge	1	0	1	0
Perceived personal risk of HIV	1	0	0	1
Attitude towards people with HIV	1	0	0	1
Waiting lists as a barrier to care	1	1	0	0
High costs as a barrier to care	1	0	1	0
Dental fear	1	1	0	0
Being recalled	1	1	0	0
Use of dental floss	1	1	0	0
**Need factors**	29			
Oral health	6	1	3	2

Missing no or not many teeth	6	2	4	0
Perceived oral health problems	2	1	1	0
Not holding regular dental attendance for important	0	0	1	0
Being satisfied with one's teeth appearance	1	1	0	0
Oral health problems	10	6	0	4
Dental attendance due to preventive reasons	2	1	0	1
General health status	5	1	1	3
Activities of daily living (ref.: no problems)	2	0	0	2
Problems with physical activities	2	0	0	2
Health problems	4	0	1	3
Oral pain	3	2	1	0
Decayed teeth	4	1	1	2
Need of treatment	8	2	3	3
General problems due to an oral problem	4	1	1	2
Missing all teeth	1	1	0	0
Wearing a denture	2	1	0	1
Filled teeth	3	3	0	0
Fluoride	1	0	0	1
Periodontal pocket measures	1	0	0	1
Eating fruit	1	1	0	0
Mental health (ref.: good)	1	0	0	1
Eating vegetables	1	0	0	1
Smoking	2	0	1	1
Place of sick care	1	0	0	1
Normal BMI	1	1	0	0
**Psychosocial factors**				
---	---	---	---	---

4
